# Association between triglyceride glucose index and obstructive sleep apnea risk in Korean adults: a cross-sectional cohort study

**DOI:** 10.1186/s12944-020-01358-9

**Published:** 2020-08-08

**Authors:** Hyeon Hui Kang, Sei Won Kim, Sang Haak Lee

**Affiliations:** 1 Division of Pulmonary, Critical Care and Sleep Medicine, Ulsan University Hospital, University of Ulsan College of Medicine, Ulsan, Republic of Korea; 2 Division of Pulmonary, Critical Care and Sleep Medicine, Eunpyeong St. Mary’s Hospital, The Catholic University of Korea, Seoul, Republic of Korea

**Keywords:** Triglyceride glucose index, Insulin resistance, Obstructive sleep apnea, Apnea-hypopnea index, Obesity, Oxygen saturation

## Abstract

**Background:**

Triglyceride glucose (TyG) index is a reliable marker of insulin resistance, which is linked to obstructive sleep apnea (OSA). However, the relationship between TyG index and OSA has not been adequately assessed. This study aimed to evaluate the association between TyG index and OSA.

**Methods:**

TyG index was assessed in 180 (mean age: 48.6 ± 13.8 years; 73.9% male) consecutive Korean adults with suspected OSA admitted to the sleep clinic at St. Paul’s Hospital between 2010 and 2012. The occurrence of more than 5 apnea-hypopnea index (AHI) events/h was used to define OSA. TyG index was calculated using the following equation: In [fasting triglycerides (mg/dL) × fasting glucose (mg/dL)/2]. All participants were grouped according to TyG index tertiles. Multivariate logistic regression analysis was used to determine factors associated with increased OSA risk.

**Results:**

The overall prevalence of OSA in study participants was determined to be 83.9%. The prevalence of OSA increased (I [lowest]: 71.6%; II: 88.7%; III [highest]: 91.4%), and lowest peripheral oxygen saturation (SpO_2_) levels decreased (I: 83.3 ± 8.5%; II: 79.9 ± 8.7%; III: 79.0 ± 8.3%), as TyG index tertile increased (*P* < 0.05). TyG index was correlated with AHI (*r* = 0.179) and lowest SpO_2_ (*r* = − 0.188) (*P* < 0.05, respectively). Univariate linear regression analysis revealed an association between TyG and AHI (β = 10.084; *P* = 0.016). Multivariate logistic regression analysis showed that TyG index (odds ratio [OR]: 3.348; 95% confidence interval [CI]: 1.081–10.372), age ≥ 55 years (OR: 5.426; 95% CI: 1.642–17.935), and obesity (OR: 3.801; 95% CI: 1.468–9.842) were associated with increased OSA risk (all *P* < 0.05). The optimal TyG index cut-off value for predicting OSA was 8.83 (sensitivity: 61.6%; specificity: 69.0%; area under the curve: 0.688; *P* = 0.001). The predictive value of the OSA cut-off value improved when age ≥ 55 years and obesity were considered.

**Conclusion:**

Increased TyG index was independently associated with increased OSA risk.

## Background

Sleep-disordered breathing is a major health problem, and obstructive sleep apnea (OSA), in particular, is an important medical condition. The prevalence of OSA in the general population is estimated to be 9 to 38%, and OSA is more common in males, the elderly, and obese individuals relative to the general population [1]. Obstructed breathing contributes to arousal, sympathetic activation, and oxygen desaturation in OSA. Therefore, OSA increases risk of cardiovascular (CV) morbidity and mortality [2, 3].

Patients with OSA often display clinical features similar to those of metabolic syndrome, which frequently involves insulin resistance (IR) [4, 5]. A number of previous studies reported that IR adversely affects clinical outcomes [6–8]. Recently, triglyceride glucose (TyG) index has been suggested as a reliable marker for IR [9–11]. However, data analyzing an association between TyG index and OSA remain insufficient. In the present study, we aimed to evaluate the association between TyG index and OSA in adults with suspected OSA.

## Methods

### Study design and subjects

We retrospectively analyzed clinical characteristics of 180 consecutive patients referred to the sleep clinic for polysomnography examinations at St. Paul’s Hospital between January 2010 and October 2012. All patients displayed at least one symptom suggesting OSA, including snoring, excessive daytime sleepiness, witnessed apneic incidents, and nocturnal choking. Overnight polysomnographic examination using a Somnostar Pro 7-3a system (Cardinal Health, Inc., Dublin, OH, USA) was performed on all study participants. Electroencephalographic, electrocardiographic, electrooculographic, and electromyographic surface electrodes were used to record patient data. Oral and nasal airflow, tracheal sounds, and abdominal and thoracic movement were also simultaneously recorded. Transcutaneous peripheral oxygen saturation (SpO_2_) was continuously monitored using a pulse oximeter. Positional changes during sleep were also recorded. After data were collected using a computerized polysomnographic system, a manual scoring was performed. Sleep status was defined using Rechtschaffen and Kales criteria [12]. The criteria of the American Academy of Sleep Medicine were applied for respiratory events [13]. Airflow reduction of ≥ 90% of baseline values for ≥ 10 s was defined as apnea. Events involving ≥ 30% airflow reduction for ≥ 10 s accompanied by a ≥ 3% drop in oxygen saturation or arousal were defined as hypopnea. Apnea-hypopnea index (AHI) was defined as the number of apnea and hypopnea events per hour that occurred during sleep. The occurrence of ≥ 5 AHI events/h was used to determine OSA. Among patients with OSA, the severity was defined as mild (5–15 AHI events), moderate (16–30 AHI events), and severe (> 30 AHI events) based on the number of events per night of sleep [14]. All blood samples were obtained after at least 8 h fasting. TyG index was determined using the following equation: ln [fasting triglycerides (mg/dL) × fasting glucose (mg/dL)/2]. Body mass index (BMI) was defined as the patient’s weight (kg)/height^2^ (m^2^). Participants with a BMI of ≥ 25 kg/m^2^ were considered obese as specified by cut-off values for Asian individuals. Hypertension was defined as systolic/diastolic blood pressure (BP) ≥ 140/90 mmHg, anti-hypertensive medication use, or a previous hypertension diagnosis. Diabetes was defined as fasting glucose ≥ 126 mg/dL, anti-diabetic medication use, or a previous diabetes diagnosis. Dyslipidemia was defined as total cholesterol ≥ 240 mg/dL, triglyceride ≥ 150 mg/dL, high-density lipoprotein cholesterol (HDL-C) ≤ 40 mg/dL, low-density lipoprotein cholesterol (LDL-C) ≥ 130 mg/dL, or lipid lowering medication use. Excessive daytime sleepiness was defined as Epworth sleepiness score (ESS) values > 10 [15]. The institutional review board approved the protocol used in the study (approval number: PC14OISI0059), and all participants provided written informed consent.

### Statistical analysis

Categorical and continuous variables are presented as number (percentage) and mean ± standard deviation, respectively. One-way analysis of variance was used to compare continuous variables. The χ^2^- test or Fisher’s exact test was used to compare categorical variables. Pearson’s correlation test was used to evaluate the correlation between variables. Univariate linear regression analysis was used to assess the association between clinical variables and AHI. Univariate and multivariate logistic regression analyses were used to evaluate independent risk factors for OSA. In logistic regression models, variables with *P* values < 0.05 in univariate regression analysis were regarded as confounding variables and were entered into multivariate regression analysis. To determine an optimal cut-off value of TyG index for predicting OSA, receiver operating characteristic (ROC) curve analysis with Youden index was used. Then, we compared the significance of this cut-off value to predict OSA considering other independent risk factors together. SAS version 9.1.3 (SAS Institute Inc., Cary, NC, USA) software was used in all statistical analyses. *P* values < 0.05 were considered significant.

### Results

Baseline characteristics of all study participants are included in Table 1. The mean age of the participants (133 males, 73.9%) was 48.6 ± 13.8 y. Mean BMI was 26.4 ± 4.1 kg/m^2^, and the prevalence of hypertension, diabetes, dyslipidemia, and obesity in the study participants was 78.3, 27.8, 72.8, and 59.4%, respectively. The prevalence of alcohol consumption and the percentage of patients with a history of smoking were 61.1 and 37.2%, respectively. The mean AHI value determined for all study participants was 31.6 ± 28.3/h. The overall prevalence of OSA (*n* = 151) was 83.9%, and the prevalences of mild (*n* = 42), moderate (*n* = 36), and severe (*n* = 73) OSA were 23.3, 20.0, and 40.6%, respectively.

**Table 1:** Baseline characteristics

Characteristics	Total (*n* = 180)	No OSA (*n* = 29)	OSA (*n* = 151)	*P*
Age, years	48.6 ± 13.8	42.5 ± 12.1	49.8 ± 13.8	0.009
Male, n (%)	133 (73.9)	16 (55.2)	117 (77.5)	0.012
Systolic BP, mmHg	129.1 ± 11.7	125.5 ± 14	129.9 ± 11.2	0.064
Diastolic BP, mmHg	88.6 ± 11.1	85.5 ± 12.5	89.3 ± 10.7	0.096
BMI, kg/m^2^	26.4 ± 4.1	23.5 ± 3.1	26.9 ± 4.1	< 0.001
Obesity, n (%)	107 (59.4)	8 (27.6)	99 (65.6)	< 0.001
Alcohol consumption, n (%)	110 (61.1)	14 (48.3)	96 (63.6)	0.122
Smoking, n (%)	67 (37.2)	5 (17.2)	62 (41.1)	0.015
Hypertension, n (%)	141 (78.3)	18 (62.1)	123 (81.5)	0.020
Diabetes, n (%)	50 (27.8)	5 (17.2)	45 (29.8)	0.167
Dyslipidemia, n (%)	131 (72.8)	17 (58.6)	114 (75.5)	0.061
ESS	9.4 ± 4.3	8.5 ± 4.0	9.5 ± 4.3	0.222
ESS > 10, n (%)	66 (36.7)	7 (24.1)	59 (39.1)	0.126
Mean SpO_2_ (%)	92.4 ± 4.3	96.1 ± 1.3	91.6 ± 4.3	< 0.001
Lowest SpO_2_ (%)	80.8 ± 8.7	90.6 ± 2.7	78.9 ± 8.1	< 0.001
AHI (/h)	31.6 ± 28.3	1.9 ± 1.5	37.3 ± 27.4	< 0.001
Sleep period time (min)	414.8 ± 52.1	418.8 ± 89.6	414.0 ± 41.7	0.779
Total Sleep time (min)	347.3 ± 67.8	353.0 ± 86.6	346.1 ± 63.8	0.619
Sleep efficiency (%)	81.3 ± 14.5	79.4 ± 18.0	81.7 ± 13.8	0.452
Sleep latency (min)	13.1 ± 28.5	22.3 ± 58.8	11.3 ± 17.4	0.057
Total cholesterol, mg/dL	186.3 ± 39.7	176.9 ± 32.0	188.1 ± 40.8	0.162
Triglyceride, mg/dL	163.2 ± 94.0	128.2 ± 58.0	170.6 ± 98.3	0.026
HDL-C, mg/dL	47.6 ± 11.5	51.4 ± 14.6	46.9 ± 10.7	0.055
LDL-C, mg/dL	113.6 ± 35.6	107.4 ± 28.2	114.8 ± 36.8	0.307
Glucose, mg/dL	103.1 ± 21.0	96.1 ± 14.5	104.4 ± 21.8	0.049
TyG index	8.91 ± 0.50	8.62 ± 0.46	8.97 ± 0.49	< 0.001

Values are given as the mean ± standard deviation or number (%)

*AHI* Apnea-hypopnea index, *BMI* Body mass index, *BP* Blood pressure, *ESS* Epworth sleepiness scale, *HDL-C* High-density lipoprotein cholesterol, *LDL-C* Low-density lipoprotein cholesterol, *OSA* Obstructive sleep apnea, *SpO*_*2*_ Peripheral oxygen saturation, *TyG* Triglyceride glucose

**Fig. 1:**
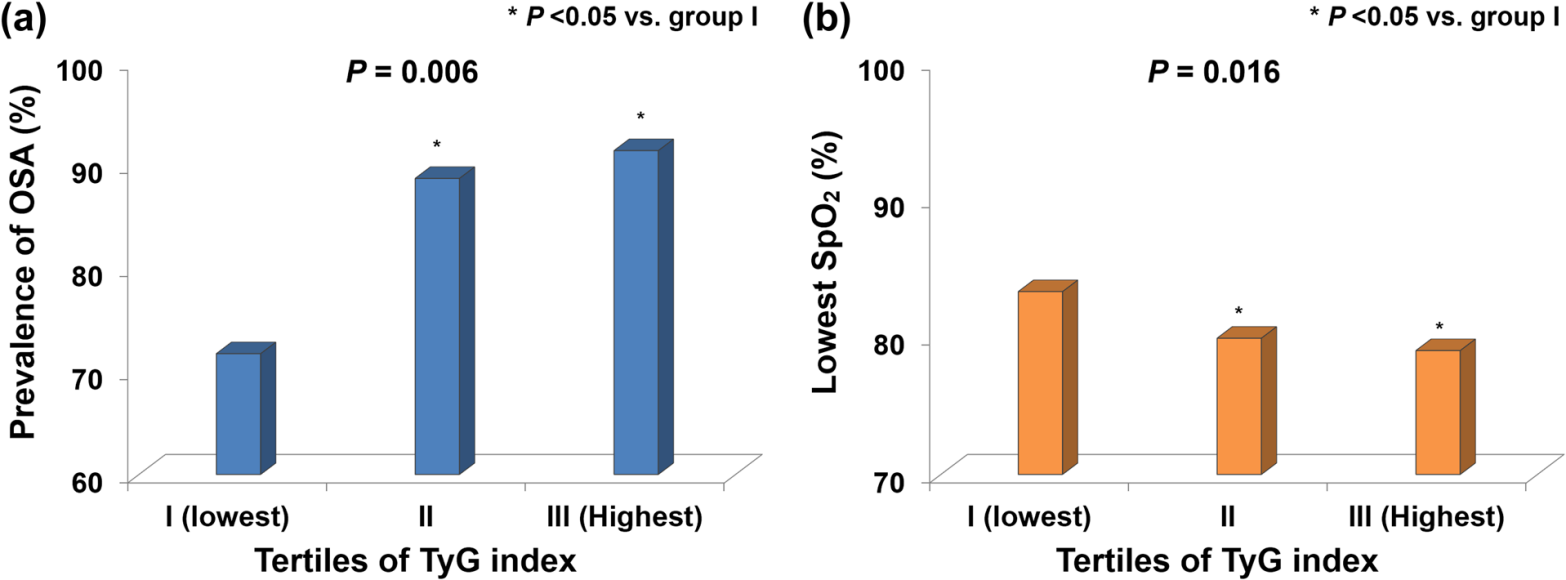
Comparison of (**a**) OSA prevalence and (**b**) the lowest SpO_2_ levels determined according to TyG index tertiles

**Fig. 2:**
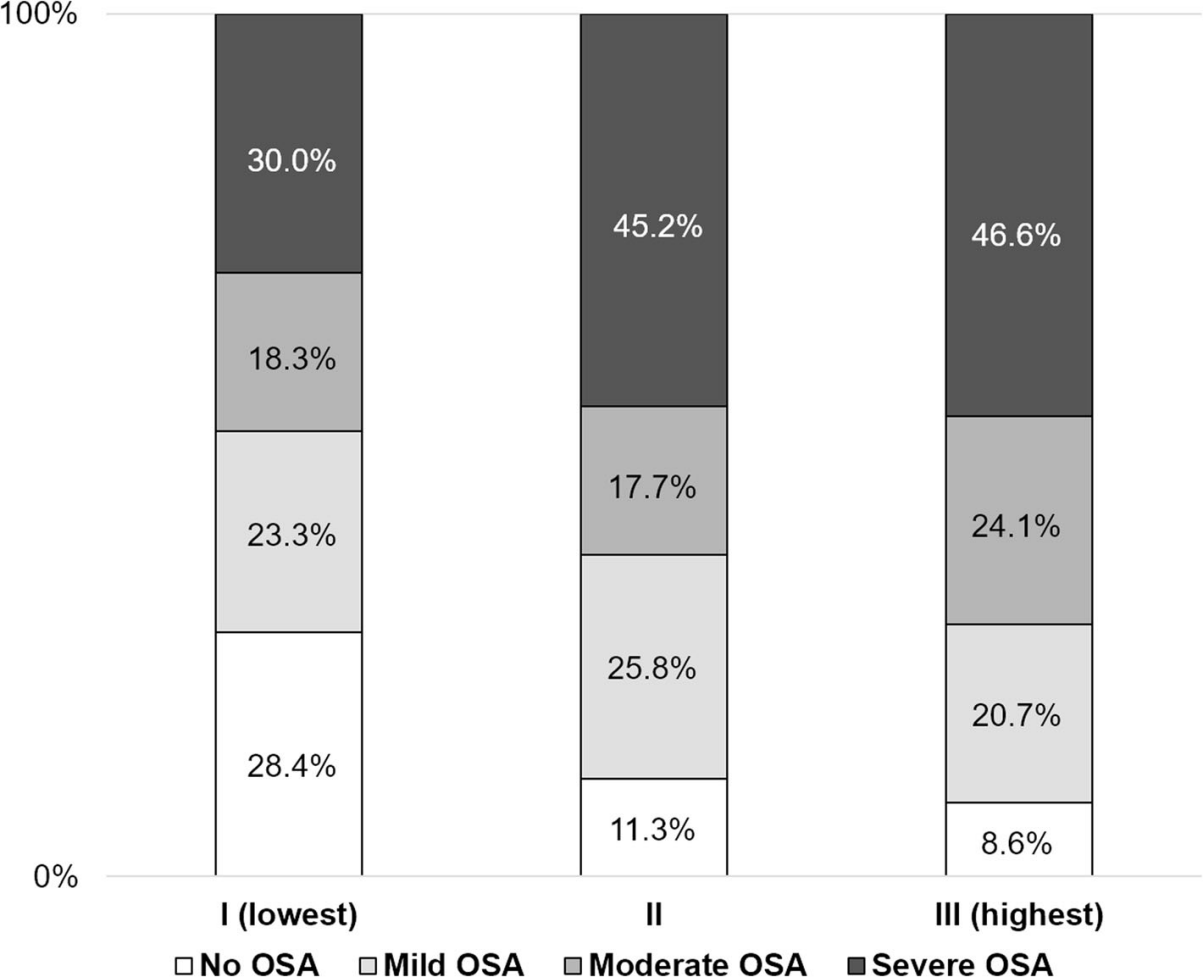
Composition of the presence and severity of OSA according to TyG index tertiles

All participants were divided into three groups based on TyG index tertiles. Patients with TyG indexes of 7.60–8.72, 8.73–9.06, and 9.07–10.49 were included in groups I (lowest), II, and III (highest), respectively. Mean TyG indexes of 8.39 ± 0.23, 8.89 ± 0.11, and 9.47 ± 0.35 were determined for groups I, II, and III, respectively. The prevalence of OSA significantly increased as TyG index tertile increased (group I [lowest]: 71.6% vs. group II: 88.7% vs. group III [highest]: 91.4%; *P* = 0.006), and no significant difference was observed between group II and group III (Fig. 1a). In contrast, the SpO_2_ nadir significantly decreased as TyG index tertile increased (group I: 83.3 ± 8.5% vs. group II: 79.9 ± 8.7% vs. group III: 79.0 ± 8.3%; *P* = 0.016), and no significant difference was observed between group II and group III (Fig. 1b). The occurrence and severity of OSA associated with all TyG index tertiles are shown in Fig. 2.

**Fig. 3:**
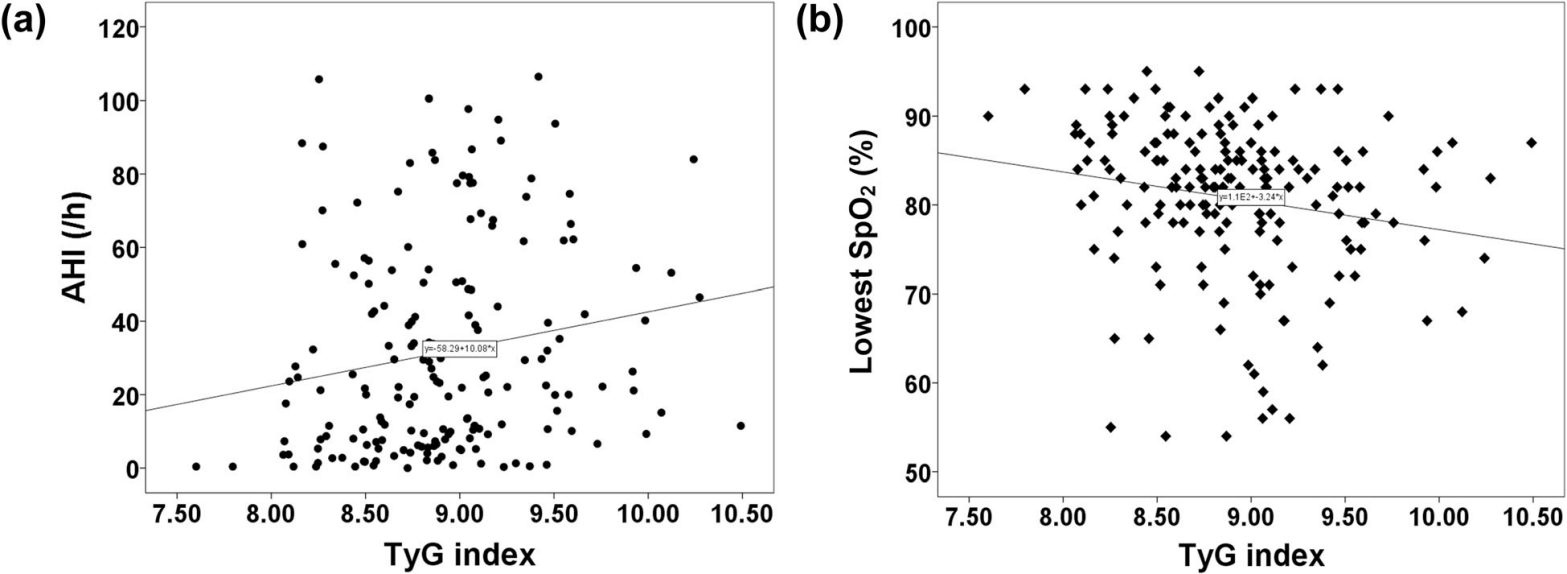
Correlation between TyG index and (**a**) AHI and (**b**) the lowest SpO_2_ value determined

Univariate linear regression analysis revealed that alcohol consumption, smoking, BMI, and TyG index were significantly associated with AHI (Table 2). TyG index correlated with increased AHI (*r* = 0.179; *P* = 0.016) and decreased SpO_2_ (*r* = − 0.188; *P* = 0.011) in all participants (Fig. 3a and b).

**Table 2:** Association between clinical variables and AHI

	β	95% CI	*P*
Age, per 1- years	0.105	−0.197–0.408	0.493
Male	9.163	−0.220–18.545	0.056
BMI, per 1-kg/m^2^	3.367	2.481–4.253	< 0.001
Alcohol consumption	11.796	3.435–20.158	0.006
Smoking	14.326	5.977–22.675	0.001
TyG index, per 1-unit	10.084	1.898–18.269	0.016

*BMI* Body mass index, *CI* Confidence interval, *TyG* Triglyceride glucose

Univariate logistic regression analysis showed that male sex (odds ratio [OR]: 2.796; 95% confidence interval [CI]: 1.225–6.383; *P* = 0.015), age ≥ 55 years (OR: 3.790; 95% CI: 1.255–11.449; *P* = 0.018), obesity (OR: 4.998; 95% CI: 2.071–12.058; *P* < 0.001), history of smoking (OR: 3.344; 95% CI: 1.210–9.242; *P* = 0.020), and elevated TyG index (OR: 5.130; 95% CI: 1.922–13.691; *P* = 0.001) were associated with OSA. Further, multivariate logistic regression analysis was used to show that age ≥ 55 years (OR: 5.426; 95% CI: 1.642–17.935; *P* = 0.006), obesity (OR: 3.801; 95% CI: 1.468–9.842; *P* = 0.006), and elevated TyG index (OR: 3.348; 95% CI: 1.081–10.372; *P* = 0.036) were independently associated with OSA (Table 3).

**Table 3:** Clinical variables and the risk of OSA

	Univariate			Multivariate		
Variables	Coefficient (95% CI)	OR (95% CI)	*P*	Coefficient (95% CI)	OR (95% CI)	*P*
Age ≥ 55 years	1.332 (0.227–2.438)	3.790 (1.255–11.449)	0.018	1.691 (0.496–2.887)	5.426 (1.642–17.935)	0.006
Male	1.028 (0.203–1.854)	2.796 (1.225–6.383)	0.015	0.776 (−0.200–1.752)	2.173 (0.819–5.767)	0.119
Obesity	1.609 (0.728–2.490)	4.998 (2.071–12.058)	< 0.001	1.335 (0.384–2.287)	3.801 (1.468–9.842)	0.006
Alcohol consumption	0.626 (−0.174–1.426)	1.870 (0.840–4.163)	0.125			
Smoking	1.207 (0.191–2.224)	3.344 (1.210–9.242)	0.020	0.718 (−0.429–1.866)	2.051 (0.651–6.460)	0.220
TyG index, per 1-unit	1.635 (0.653–2.617)	5.130 (1.922–13.691)	0.001	1.208 (0.078–2.339)	3.348 (1.081–10.372)	0.036

*BMI* Body mass index, *CI* Confidence interval, *OR* Odds ratio, *OSA* Obstructive sleep apnea, *TyG* Triglyceride glucose

**Fig. 4:**
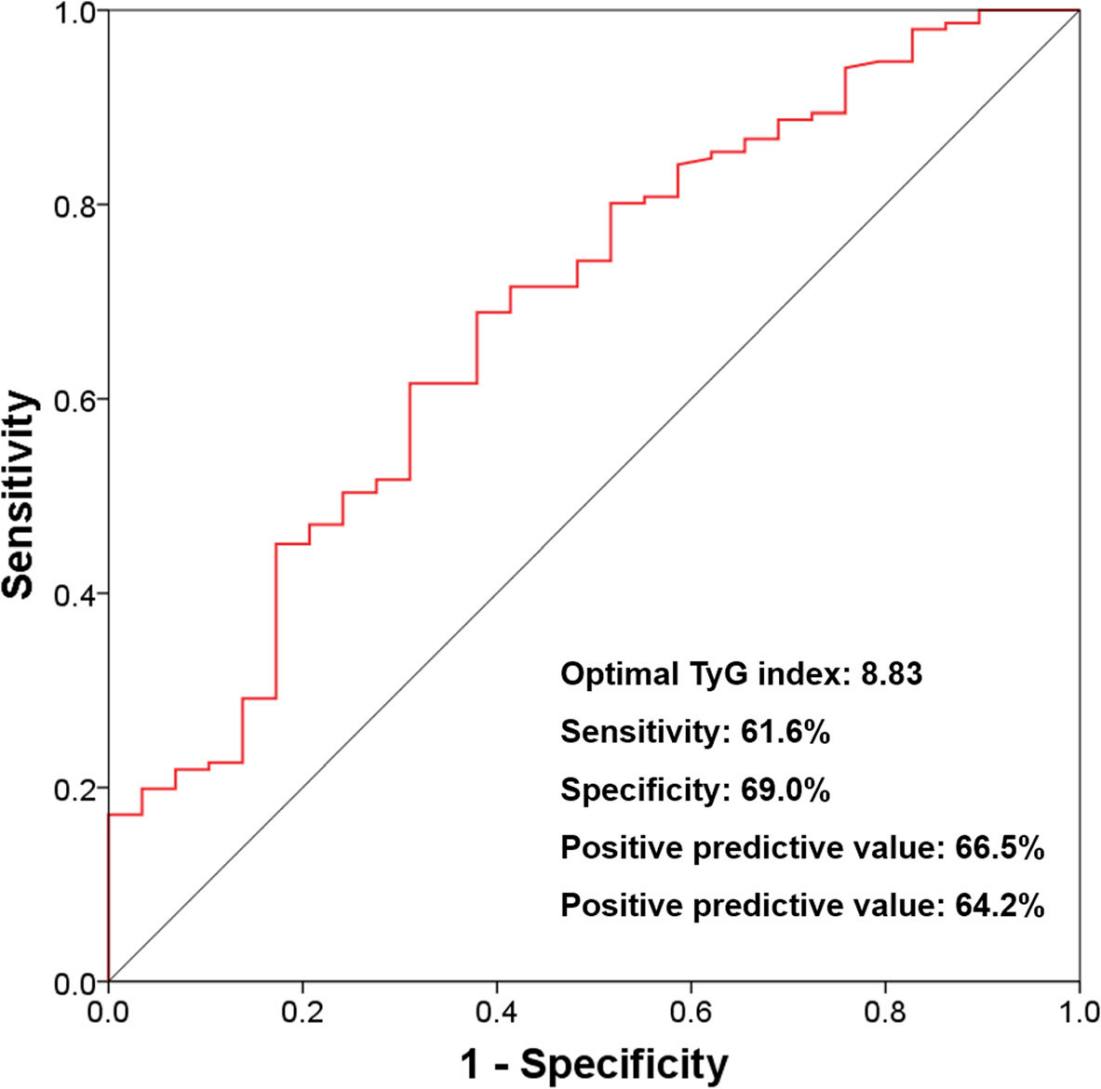
ROC curve used to determine the optimal TyG index cut-off value for predicting OSA

ROC curve analysis showed that the optimal TyG index cut-off value used for predicting the presence of OSA, as determined via Youden index, was 8.83 (sensitivity: 61.6%; specificity: 69.0%; area under the curve [AUC]: 0.688; 95% CI: 0.582–0.794; *P* = 0.001) (Fig. 4). The usefulness of TyG indexes greater than 8.83 for predicting OSA significantly improved after considering whether patients were aged ≥ 55 years (TyG ≥ 8.83 vs. TyG ≥ 8.83 mg/dL for patients aged ≥ 55 years; AUC: 0.618 vs. 0.703; *P* = 0.002) and whether patients were aged ≥ 55 years and obese (TyG ≥ 8.83 vs. TyG ≥ 8.83 in patients aged ≥ 55 years and obese; AUC: 0.618 vs. 0.772; *P* < 0.001) (Table 4).

**Table 4:** Comparison of ROC models related to the cut-offs of TyG index for predicting OSA

ROC Models	AUC (95% CI)
TyG index ≥ 8.83	0.618 (0.520–0.716)
TyG index ≥ 8.83 with age ≥ 55 years	0.703 (0.600–0.806)*
TyG index ≥ 8.83 with age ≥ 55 years and obesity	0.772 (0.673–0.871)^†^

*AUC* Area under the curve, *CI* Confidence interval,

OSA Obstructive sleep apnea, *ROC* Receiver operating characteristic

*P = 0.002 vs. TyG index ≥ 8.83. ^†^*P* < 0.001 vs. TyG index ≥ 8.83

## Discussion

To the best of our knowledge, this is the first study to evaluate the association between TyG index and OSA risk in adults with suspected OSA. In the present study, elevated TyG index was independently associated with an increased OSA risk. We identified the optimal cut-off value that indicates the limits of its usefulness for predicting OSA in the population assessed. The predictive value of the cut-off was significantly improved after considering other independent risk factors of OSA.

IR has been implicated in metabolic syndrome and CV disease pathogenesis [6–8]. Also, previous studies have revealed a close relationship between IR and sleep-disordered breathing. Punjabi et al. [16] reported that sleep-disordered breathing was associated with impaired glucose tolerance and IR, independent of obesity. Ip et al. [17] also reported that obesity was the major determinant of IR, and despite adjusting for obesity and other confounding factors associated with IR, minimum oxygen saturation and AHI were determined to be significant determinants of fasting insulin level and homeostatic model assessment of insulin resistance (HOMA-IR) index. The HOMA-IR has been used as a marker of IR, but insulin level must be measured to determine HOMA-IR [18]. Recent data have shown the close relationship between HOMA-IR and TyG index [10, 11, 19, 20]. However, little is known regarding the significance of TyG index in the risk of OSA despite the close relationship between IR and OSA.

Drager et al. [21] reported that OSA was independently associated with factors important for diagnosing metabolic syndrome, including triglyceride (OR: 3.26; 95% CI: 1.47–7.21) and glucose (OR: 2.31; 95% CI: 1.12–4.80) levels. Moreover, Meszaros et al. [22] recently reported that the co-occurrence of OSA and hypertriglyceridemia is affected by genetics, and heritable factors might play a crucial role in dyslipidemia pathogenesis in OSA. These results indicated that there may be a close relationship between TyG index and OSA. In the present study, despite the relatively small sample size, a significant positive correlation between TyG index and AHI was observed, although the individual lipid profile did not significantly correlate with AHI (Supplementary table 1). In addition, TyG index was negatively correlated with decreased SpO_2_. Similarly, TyG index also had an inverse association with mean SpO_2_ value **(**Supplementary figure 1**)**. After controlling for confounding factors, TyG index, together with age (≥ 55 years) and obesity, was determined to be associated with OSA risk.

Park et al. [23] recently reported the predictive significance of TyG index for subclinical coronary artery disease in patients with no traditional CV risk factors; the mean TyG index was 8.31 ± 0.46. In the present study, the mean TyG index was higher than that reported by Park et al. This suggests that patients with suspected OSA have increased IR. Although the predictive significance of the optimal cut-off value of TyG index for OSA was poor, this was moderately improved after considering other independent risk factors. Its predictive power might be acceptable in populations with specific clinical conditions. Considering that data on the relationship between TyG index and OSA risk has been limited in sleep medicine, the present study has the potential to provide important information applicable to the adult population at high risk for OSA.

### Study strengths and limitations

The present study had some limitations. First, this study had an observational design. Thus, clinical factors not considered could influence the study results. Second, the population considered had an unbalanced sex distribution; more males than females were referred to sleep clinics within the study period. Third, we were unable to consider HOMA-IR, because there was a paucity of data regarding insulin level for the population studied. However, the close relationship between HOMA-IR and TyG index has previously been well established. Fourth, we could not eliminate the possible effects of underlying medications on OSA because of the observational design of this study. Finally, considering that only patients highly suspected of having OSA were included in the present study, these results should be applied with caution to the general population. Despite these limitations, these findings revealed that TyG index may be used to predict OSA in Korean adults with suspected OSA.

## Conclusions

In this cross-sectional, observational study, TyG index was determined to be independently associated with risk of OSA in Korean adults admitted to the sleep clinic. Further, prospective, large-scale, and longitudinal studies will be needed to confirm the value of the TyG index for predicting OSA.

## Supplementary information

**Additional file 1: Table S1.** Association of individual lipid profile with AHI. **Figure S1.** Association between TyG index and mean SpO_2._

## Data Availability

The datasets used and analyzed during the current study will be provided by the corresponding author upon reasonable request.

## Abbreviations

AHI: Apnea-hypopnea index
AUC: Area under the curve
BMI: Body mass index
CV: Cardiovascular
CI: Confidence interval
ESS: Epworth sleepiness score
HDL-C: High-density lipoprotein cholesterol
HOMA-IR: Homeostatic model assessment of insulin resistance
IR: Insulin resistance
LDL-C: Low-density lipoprotein cholesterol
OR: Odds ratio
OSA: Obstructive sleep apnea
ROC: Receiver operating characteristic
SpO_2_: Peripheral oxygen saturation
TyG: Triglyceride glucose
