# Supplementary material for: Association between triglyceride glucose index and obstructive sleep apnea risk in Korean adults: a cross-sectional cohort study
Source: Lipids Health Dis. 2020 Aug 8;19:182. doi: 10.1186/s12944-020-01358-9 (PMC7414547; doi:10.1186/s12944-020-01358-9)

**Supplementary table 1.** Association of individual lipid profile with AHI

|  | β | 95% CI | *P* |
| --- | --- | --- | --- |
| Total cholesterol, mg/dL | 0.076 | -0.029–0.181 | 0.153 |
| Triglyceride, mg/dL | 0.034 | -0.010–0.078 | 0.128 |
| HDL-C, mg/dL | -0.241 | -0.602–0.121 | 0.191 |
| LDL-C, mg/dL | 0.112 | -0.004–0.228 | 0.059 |
| Glucose, mg/dL | 0.180 | -0.018–0.377 | 0.074 |

CI = confidence interval; HDL-C = high-density lipoprotein cholesterol; LDL-C = low-density lipoprotein cholesterol.

**Supplementary figure 1.** Association between TyG index and mean SpO_2_


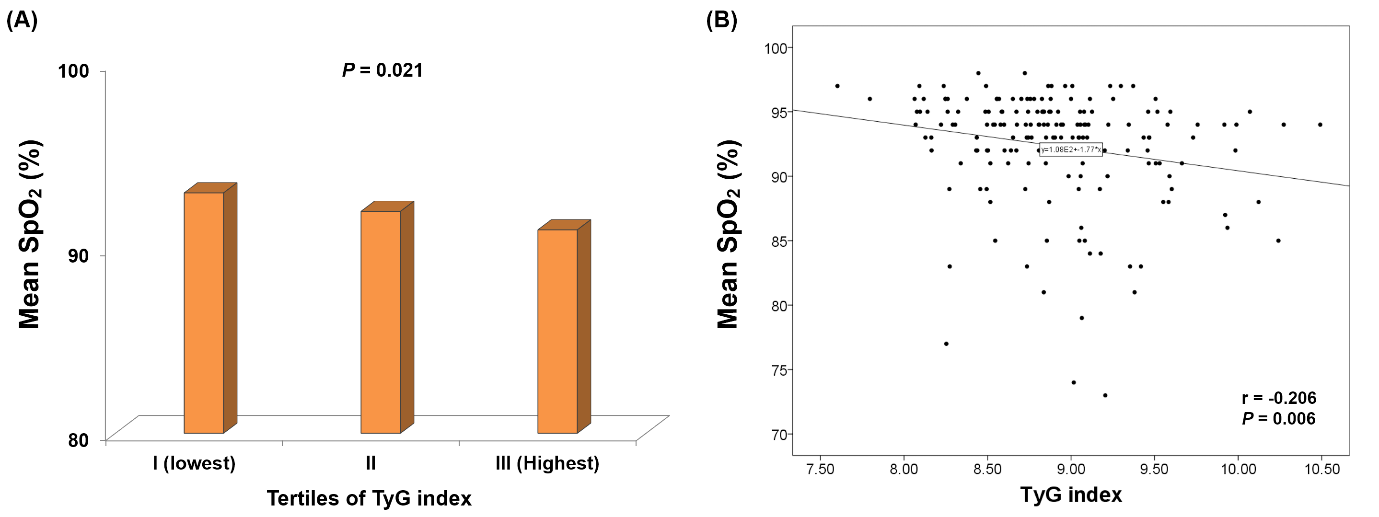

Supplement: Supplementary file 1 — Additional file 1: Table S1. Association of individual lipid profile with AHI. Figure S1. Association between TyG index and mean SpO2. [file 12944_2020_1358_MOESM1_ESM.docx]
